# MiRNA-Nanofiber, the Next Generation of Bioactive Scaffolds for Bone Regeneration: A Review

**DOI:** 10.3390/mi12121472

**Published:** 2021-11-29

**Authors:** Davood Kharaghani, Eben Bashir Kurniwan, Muhammad Qamar Khan, Yuji Yoshiko

**Affiliations:** 1 Department of Calcified Tissue Biology, Graduate School of Biomedical and Health Sciences, Hiroshima University, 1-2-3 Kasumi, Minami-ku, Hiroshima 734-8553, Japan; yyuji@hiroshima-u.ac.jp; 2 School of Dentistry, Hiroshima University, 1-2-3 Kasumi, Minami-ku, Hiroshima 734-8553, Japan; ebenbashir99@gmail.com; 3 Nanotechnology Research Lab, Department of Textile and Clothing, National Textile University, Karachi Campus, Karachi 74900, Pakistan; drqamar@ntu.edu.pk

**Keywords:** bone regeneration, bone formation, nanofiber, miRNA delivery, scaffold

## Abstract

Scaffold-based bone tissue engineering has been introduced as an alternative treatment option for bone grafting due to limitations in the allograft. Not only physical conditions but also biological conditions such as gene expression significantly impact bone regeneration. Scaffolds in composition with bioactive molecules such as miRNA mimics provide a platform to enhance migration, proliferation, and differentiation of osteoprogenitor cells for bone regeneration. Among scaffolds, fibrous structures showed significant advantages in promoting osteogenic differentiation and bone regeneration via delivering bioactive molecules over the past decade. Here, we reviewed the bone and bone fracture healing considerations for the impact of miRNAs on bone regeneration. We also examined the methods used to improve miRNA mimics uptake by cells, the fabrication of fibrous scaffolds, and the effective delivery of miRNA mimics using fibrous scaffold and their processes for bone development. Finally, we offer our view on the principal challenges of miRNA mimics delivery by nanofibers for bone tissue engineering.

## 1. Introduction

Bone loss or damage can result from various causes, including degenerative diseases, fractures, and surgeries. Though bone can repair itself, the regeneration of damaged bone needs to be stimulated in many cases when complete bone regeneration cannot occur [1]. MiRNAs have been shown to alter expression levels in bone regeneration significantly. MiRNA, a class of non-coding RNA, plays a vital role in gene expression by targeting mRNAs to destabilize mRNAs and/or inhibit protein translation [2,3,4]. In recent years, miRNA mimic delivery has been investigated to treat various diseases and conditions, including cancer and neurodegenerative disorders [5]. 

A wide range of advanced scaffolds have been developed in the last decade for bone regeneration. Scaffold-based DDS (Table S1) for bone tissue engineering have evolved as an interdisciplinary approach to designing platforms from the perspective of both materials and functions. These platforms provide structures, helping reorganize the tissues of defective or lytic bone lesions [6]. Until recently, emphasis was only placed on microporous framework structures that can mimic physical features of ECM and support cell adhesion, migration, and morphogenesis [7]. Therefore, many scaffolds and methods are involved in the preparation of 3D micropores constructions. Amongst porous scaffolds, NFs are prominent in bone tissue engineering due to their ability to promote adhesion and osteogenic differentiation of bone borrow MSCs [8]. NFs are better candidates than hydrogels for drug delivery systems due to their high surface-to-volume ratio and porosity. A notable feature of NFs is their ability to promote the transfer of drugs and waste products and making them an ideal scaffold for both DDS and tissue engineering [9]. 

The field of bone tissue engineering has recently been utilizing DDS for delivering functional molecules, including bioactive signal molecules [10]. Over the last decade, prominence has been placed on signaling molecules like non-coding RNAs, to improve the biocompatibility and biodegradation of scaffolds, and control cell adhesion and differentiation [8]. The miRNAs that regulate gene expression at the post-transcriptional level are evolutionally involved in several transcription factors and cytokines. It has been proven that, besides the architecture of the scaffolds that have been used for bone tissue engineering, miRNA mimics provide a link between the scaffold and activation of osteogenic markers. In the case of bone regeneration, a combination of NFs and miRNA mimic enhanced bone formation through cell signaling. For example, loading miRNA-22 and miRNA-126 mimics into PCL NFs could promote cell viability and osteogenic differentiation of human iPSCs with increased osteoblast marker gene expression, including *RUNX2*, *BGLAP*, *ALPL*, and *SPARK* [11].

**Figure 1:**
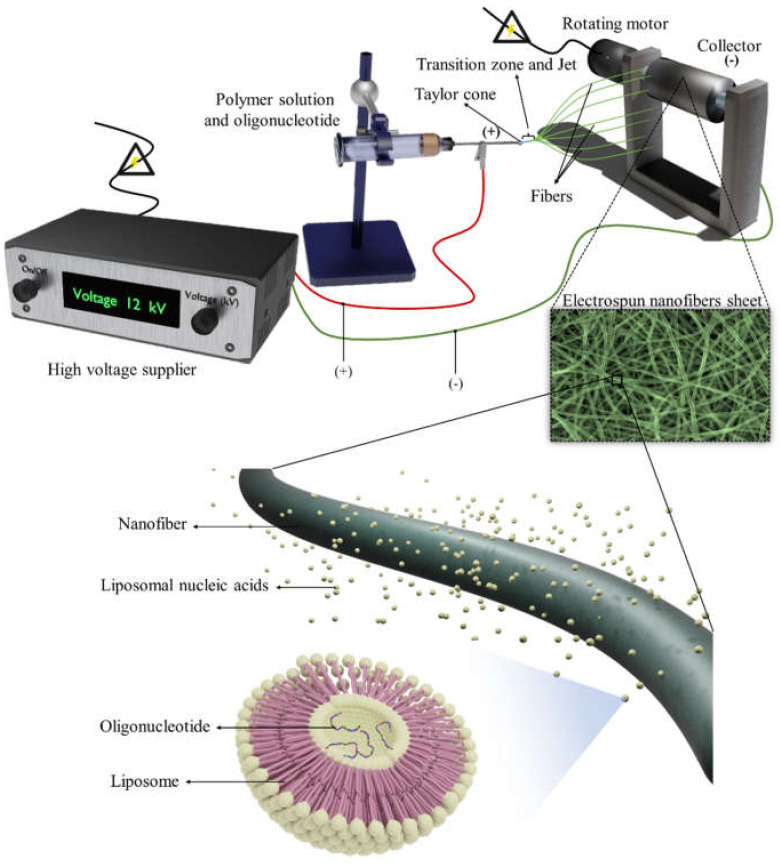
An illustration of the electrospinning system for NF formation. Electrospinning involves supplying an electric charge to a polymer solution in a syringe and subsequently stretching polymer droplets to form zones termed the Taylor cone and Jet, respectively, at the tip of the needle to form fibers. The produced fiber accumulates on a collector under the influence of electrostatic repulsion between the reference (polymer solution) and counter electrode (collector). Nanofibers can deliver liposomal synthetic oligonucleotides.

To explain the application of fibrous scaffolds as miRNA mimic carriers to bone tissue engineering (see Figure 1), we describe fracture healing aspects of the impact of miRNAs on bone regeneration and methods to deliver miRNAs mimic into target cells. Moreover, we describe the impact of NF scaffolds to deliver miRNA mimics for bone tissue engineering. Finally, we discuss fundamental challenges and prospects in the application of a complex of NF and miRNA. 

## 2. Bone Healing and MiRNAs Expression

**Figure 2:**
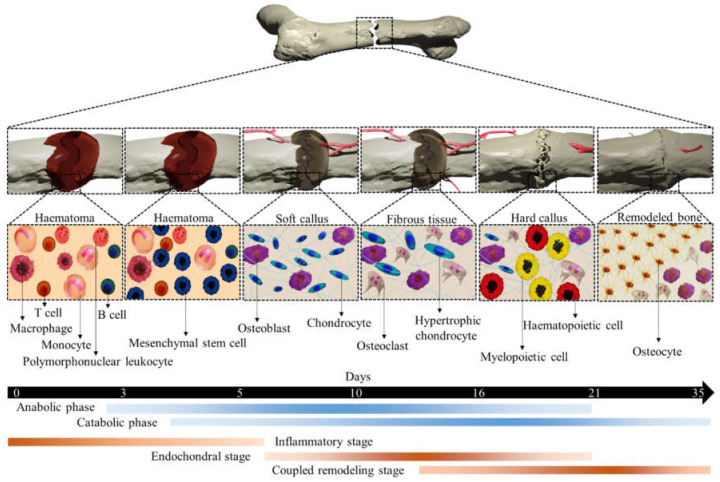
An illustration of different stages of typical fracture healing. The metabolic phases (blue bars) overlapped with biological stages (brown bars) for 35 days (black bars). The three biological stages of bone fracture healing are inflammatory, endochondral bone formation, and coupled remodeling. The time scale denoted is based on the primary cell types present at each stage of healing of a mouse closed femur fracture. This figure was recreated from 2D to 3D with permission from reference [14], Copyright 2021, Springer Nature.

The adult bone adults constantly remodel itself, responding to both various physiological and pathological demands, such as repairing bones weakened by macro- and microdamage. Bone regeneration is a complex process, involving both bone remodeling and cellular processes. In both endochondral and intramembranous ossification, a number of complex gene expressions are involved in reaching the remodeled bone. Over the past several decades, our understanding of either process has improved enormously [12]. In both cases, MSCs migrate to the damaged area and increase growth factors and cytokines levels followed by differentiation into chondrocytes (endochondral ossification) or osteoblasts (intramembranous ossification). After an injury, a cartilaginous callus forms at the injury site, which provides intermediate stabilization to the fracture segment. This cartilage callus subsequently becomes vascularized, remodeled, and finally will be replaced by bone. Conversely, in intramembranous ossification, MSCs differentiate directly into osteoblasts in a highly stabilized fracture healing without a prolonged cartilaginous intermediate [13]. Fracture healing is an apparent example of endochondral ossification that begins with fracture-induced vascular disruption. Exposure of vascular cells triggers a coagulation cascade (thrombin activation) and the subsequent formation of hematoma, where platelets and macrophages accumulate. A local inflammatory reaction is initiated to release inflammatory mediators from the fracture hematoma, causing vascular permeability and inflammatory cell migration, as shown in Figure 2. These processes activate osteoclasts to resorb bone debris and fibroblasts to transform the hematoma into granulation tissue. New vasculature in the fracture site provides MSCs that transformed to the cells with osteogenic potential and formed callus. A soft and hard callus could convert to the bone via endochondral ossification and intramembranous ossification, respectively.

Currently, more than 1000 articles in the PubMed database address questions about miRNAs in bone cells, including osteoblasts and osteoclasts. The microarray data from femoral fracture of twelve-week-old male Sprague-Daweley rats showed that 317 miRNAs were expressed more highly in normal healing fractures. However, eight miRNAs include rno-miR-140-3p, rno-miR-140-5p, rno-miR-181a-5p, rno-miR181d-5p, rno-miR- rno-208b-3p, rno-miR-451a, rno-miR-743b-5p, and rno-miR-879-3p, which were highly up-regulated by filtering with a fold change of >2.0, a low coefficient variation (<50%) during 28 days, as presented [15]. Recent studies demonstrated that miR-140-3p and miR-140-5p are highly expressed in the cartilage. Mmu-miR-140-3p and mmu-miR-140-5p are involved in osteoarthritis. Mmu-miR-140-3p with targeting the *Cxcr4* and *Rala* promotes chondrogenesis and ameliorates osteoarthritis progression. Besides, the miR-140-5p with downregulating *Il1b*, *Il6*, *Mmp13*, *Smad3*, and *Hmgb1* expression, inhibit inflammation. However, miR-140-5p with the upregulation of *Dnpep*, *Tgfbr1,* and *Rala* expression promotes chondrogenesis. Moreover, expression of miR-140-5p involves the upregulation of *Hdac4* and *Smad1*, and results in the inhibition of chondrocyte hypertrophy and osteogenesis with downregulation *Tlr4*, *Bmp2,* and *Tgfbr1* genes, respectively. MiR-181a-5p as well as miR-140-3p and miR-140-5p was identified as inflammation regulator at the early stage of fracture healing by targeting *NCOA1*, *NRIP1*, and *IL1A* gens. The treatment of human MSCs by miR-181d-5p mimic indicated that downregulation of miR-181d-5p promotes osteogenic differentiation by targeting *RUNX2* through the MAPK pathway. Moreover, miR-181a-5p promotes osteoblastic differentiation via the procession of TGF-β secretion. Furthermore, both miR-181a-5p and miR-181d-5p, by upregulating *BCL2*, induce osteocyte apoptosis [16]. MiR-208b-3p was another miRNA that was expressed highly in bone fracture healing. Importantly, miR-208b-3p mimic suppressed osteoblast genesis in MC3T3-E1 and inhibited bone formation by reducing *Acvr1b* translation, thereby decreasing *Bmp2* and its downstream target *Smad1/4/5* and *Runx2* [17].

**Figure 3:**
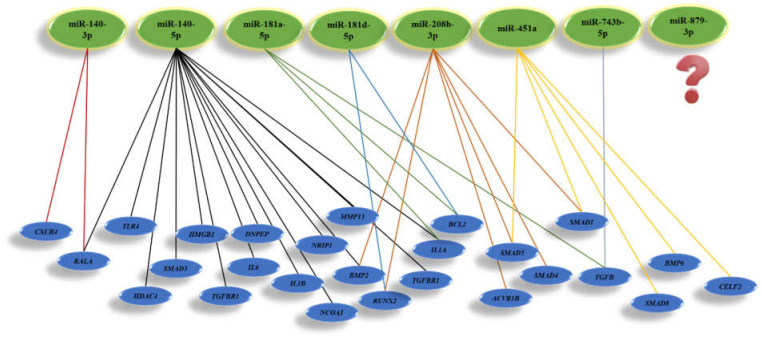
Illustration of miRNAs highly involved in bone regeneration and their target genes.

Moreover, the overexpression of mmu-miR-451a could inhibit the osteogenic differentiation of mice MSCs, accelerate bone loss via *Bmp6* signaling, and elevate bone loss by regulating *Smad1/5/8* expression [18]. Additionally, miR-451a downregulates the *CELF2*, thereby significantly increasing COX protein and inhibiting chondrocyte hypertrophy and inflammation. It is unfortunate that still, there is no significant evidence for the role of miR-743b-5p and miR-879-3p in the bone field. Possibly, miR-879-3p is only expressed in mice and rats. However, it is reported that negative expression of mmu-miR-743b-5p could enhance fibrogenesis and *Tgfb1* expression level [19]. TGFβ release from the bone matrix plays a vital role in the temporal and spatial regulation of bone remodeling during osteoclast bone resorption. Active TGFβ recruits MSCs to the bone resorption pit through the SMAD signaling pathway [20]. Moreover, activating the TGFβ pathway could promote angiogenesis in CRC cells [21]. The mi-RNAs involved in rat bone regeneration and considering their target genes applicable for human use are summarized in Figure 3. 

Intermembranous bone ossification, such as alveolar bone healing, is well studied by Anderia *Espindola Vieira* et al. [22]. It was found that intramembranous bone healing process in mice follows by tooth extraction and initiates with clot formation. Based on the results of the mice model, it appears that blood clots are gradually resorbed and replaced by granulation tissue associated with *Col1a1* and *Col1a2* expression over the course of 14 days. Moreover, they showed that growth factors and cytokines such as BMP2/4/7, FGF2, TGFβ1, VEGFA, TNFα, and IL10 were highly expressed during the first week of healing, indicating a lack of osteochondral gene markers. Unfortunately, up to now, there is no evidence related to miRNA expression in intramembranous ossification. The development of scaffolds may depend on understanding molecular signaling pathways involved in bone regeneration besides the miRNAs that are highly expressed during bone fracture healing. 

MiRNA stability is a concern when considering clinical use; for example, many miRNAs rapidly degrade at 37 °C when incubated with serum [23]. MiRNA mimics are oligonucleotides that have been chemically altered to increase their stability and affinity for target genes [24,25]. Moreover, antagomirs, synthetic antagonists of miRNA, are currently undergoing phase 1 and phase 2 clinical trials. Therefore, various miRNA mimics and antagomirs have been evaluated for bone regeneration purposes. Few articles have reviewed miRNA incorporation with hydrogel scaffolds for bone regeneration [26], but none have focused on NFs. Among the trials, several miRNAs have been loaded to hydrogels for bone regeneration, as updated and summarized in Table 1.

## 3. MiRNA Mimic Uptake by Cells

The hydrophilic nature of synthetic oligonucleotides limits their ability to penetrate the hydrophobic cellular membranes [56]. To overcome these limitations, a variety of biocompatible nanocarriers have been used to deliver miRNA mimics into target cells, including calcium phosphate, gold nanoparticles [57,58,59,60,61], and liposomes [62]. The encapsulation of miRNA mimics in cationic liposomes, which have a high affinity for cell membranes with a net negative charge, is one approach to this problem [63]. Additionally, cellular uptake is facilitated by charge interactions between the cationic lipids and the anionic oligonucleotides, which result in a net positive charge in the complex [64]. Cationic liposomes might also provoke a pro-inflammatory response by inducing Th1 cytokine expression [65]. However, the immunogenicity and cytotoxicity of liposomes are challenging for clinical applications if high and frequent dosages are required [66]. Low toxicity, tissue-specific targeting, and proper cellular uptake are critical requirements for lipid-based nanoparticles in delivery of miRNA mimics [67]. For example, siPORTTM NeoFXTM [49], HiPerFect [68], and Lipofectamine® [69] are commercially available transfection reagents, which have been extensively used to deliver miRNA mimics.

As well as liposomes, biocompatible nanoparticles such as gold [70], silica [71], and calcium phosphate [58] have been used to carry miRNAs into cells. Alkyl-thiol-terminated oligonucleotides modified in gold nanoparticles showed a high affinity for miR-145 mimic, and subsequently increased miR-145 mimic uptake by PC3 cells and MCF7 cells [70]. The mesoporous silica nanoparticles demonstrate favorable drug delivery properties due to their large surface area and pore volume, tunable particle size and pore size, and biocompatibility [72]. Mesoporous silica nanoparticles with carrying a polyarginine-peptide: nucleic acid conjugate targeting oncogenic miR-221 mimic efficiently induced apoptosis in the T98G cells [73]. The long-chain miR-34a mimic conjugates (Lc-miR34a mimic) were introduced more efficiently into PC3 cells than lc-miR-34a mimic alone, with concomitant effects on cell proliferation and migration [74]. Several carriers, including transfection reagents, have been successfully used to deliver miRNAs into various cells in vitro. However, despite their success, miRNA mimics do not possess specific affinity for pathological sites.

**Figure 4:**
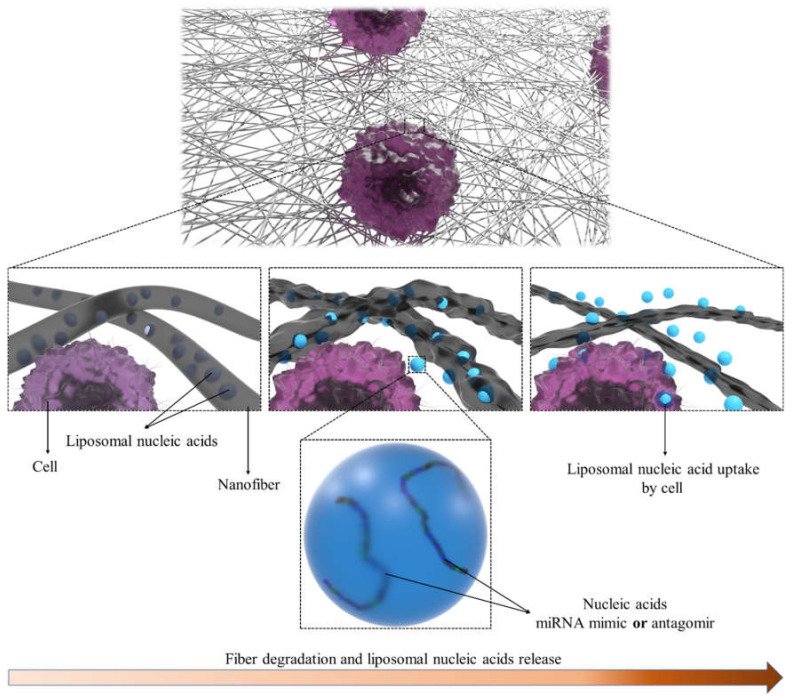
A schematic of NFs carrying miRNA mimic or antagomir-loaded liposomes. Liposomes are released as NF degrade and are transferred with their contents to target cells.

To overcome the challenges encountered in delivering miRNA mimics to target cells, several approaches have been proposed. Effective drug delivery systems accumulate and retain active ingredients locally, thereby minimizing systemic adverse effects. [75]. Since miRNAs mimic are released into the circulation, delivering them into recipient cells at appropriate spatial and temporal scales [76], they needed appropriate scaffolds to carry miRNAs for targeting bone tissue. Therefore, further studies are required to develop platforms to accumulate the miRNAs at the specific sites of interest for tissue-specific targeting of miRNAs, as shown in Figure 4. 

## 4. NFs Promote Osteogenesis

Electrospinning is an effective technique for manufacturing NFs from polymeric materials, when polymer concentration, solubility, and composition are controlled along with applied voltage, and can produce NFs with different features, including different topography, density, and fiber diameter (from a few nanometers to several micrometers) [77]. Figure 1 outlines the electrospinning technique, where a high voltage positive charge is applied to the electrostatic repulsion of the polymer solution. Droplets of positively charged polymer liquid could stretch when exposed to a negative charge, and eventually, the stretched droplets accumulated as fibers on a negatively charged metallic collector [78]. There has been considerable effort to mimic bone healing conditions with scaffolds. NFs have the excellent ability to capture drugs and complexes, such as liposome-encapsulated drugs, and release them based on parameters such as the chemical composition and structure of the fibers (e.g., the ratio of surface to volume) [75]. 

Interdisciplinary approaches have enabled us to design and develop scaffold-based cell engraftment platforms for bone tissue engineering [79]. Scaffolds with pore sizes of 100 to 300 µm and a porosity of more than 90% have been shown to enhance bone regeneration [80]. Many fabrication techniques have been introduced to facilitate the production of nanofibers from biocompatible polymers. A network of interconnected pores within highly porous microstructures have been found to promote tissue ingrowth in bone regeneration. Such 3D microporous frameworks structurally mimic the bone ECM and support cell adhesion, migration, and proliferation [81]. However, in contrast, Beom Su Kim et al. showed that PCL/SF NFs with a pore size of around 60.6 μM improved the MSCs adhesion and growth in comparison to PCL NFs, with a pore size range around 121.7 μM [82]. Recently, attention has turned to polymeric NFs, allowing scaffold designs for tissue engineering and DDS [75]. 

**Figure 5:**
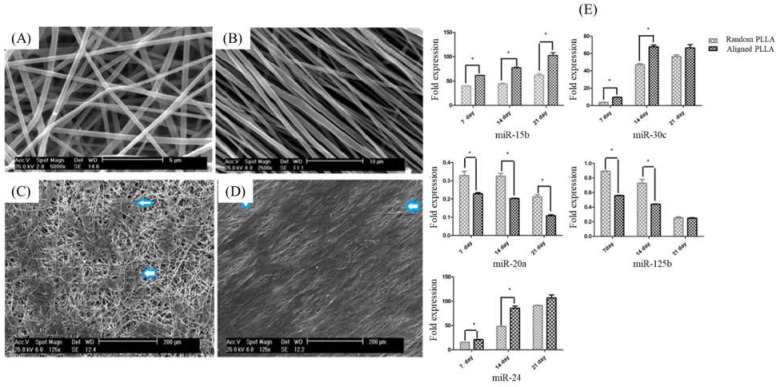
Morphology of fabricated PLLA nanofibers, randomly oriented (**A**) and aligned oriented (**B**). the morphology of adipose tissue-derived MSC adhered to random (**C**) and aligned (**D**) nanofibers. Real-time PCR analysis of miRNAs expression during osteoblast differentiation (**E**). The expression of osteoblast-specific miRNAs (miR-15b, miR-30c, miR-20a, miR-125b, and miR-24) in osteo-differentiated cells cultured on random and aligned nanofibers during 21 days of osteogenic differentiation, * *p* < 0.05. This figure was reprinted with permission from reference [86], Copyright 2021, Elsevier.

In bone, osteoblasts produce ECM principally comprising type I collagen, and subsequently contribute to ECM mineralization [12]. Hence, a successful strategy for skeletal regeneration depends on whether enough active bone- or cartilage- and bone-forming cells can be recruited and appropriately differentiated. Electrospinning is a versatile method to produce NFs with variable features of such properties as orientation, morphology, and chemical composition [83]. The potential ability of NFs assemblies to support bone regeneration must be assessed using a combination of well-established detection methods for the proliferation-differentiation of osteoprogenitor cells and matrix mineralization phase of osteogenesis: e.g., MTT for proliferation, sequential expression of osteoblast/chondrocyte marker genes, and ALP activity for differentiation and extracellular calcium deposition for mineralization. In this direction, several studies have shown that random or aligned PLLA NFs fabricated by electrospinning induced osteogenesis in MG63 cells (Human osteosarcoma cell line) [84] and MSCs from Wistar rats [85]. Furthermore, both random and aligned PLLA NFs promoted osteogenic activities in human adipose tissue-derived MSC cultures, indicating that random NFs are more effective in cell proliferation and aligned NFs can stimulate osteogenic differentiation, as shown in Figure 5 [86]. In a recent example, the topography and chemistry of electrospun poly (butylene succinate)-based NFs affected adhesion and differentiation of Saos-2 cells suggesting that an ether linkage involved in the density of hydrogen bond acceptors on the surface of NFs may contribute to osteogenic activity (expression of osteogenic markers, differentiation, and mineralization of cells) [87]. Highly porous polymeric cellulose nanofibrils can also transfer glucose, proteins, oxygen, and waste products, which lead to promoting proliferation of MG63 cells [88].

## 5. Bone Tissue Regeneration by MiRNAs Mimic-NFs

**Figure 6:**
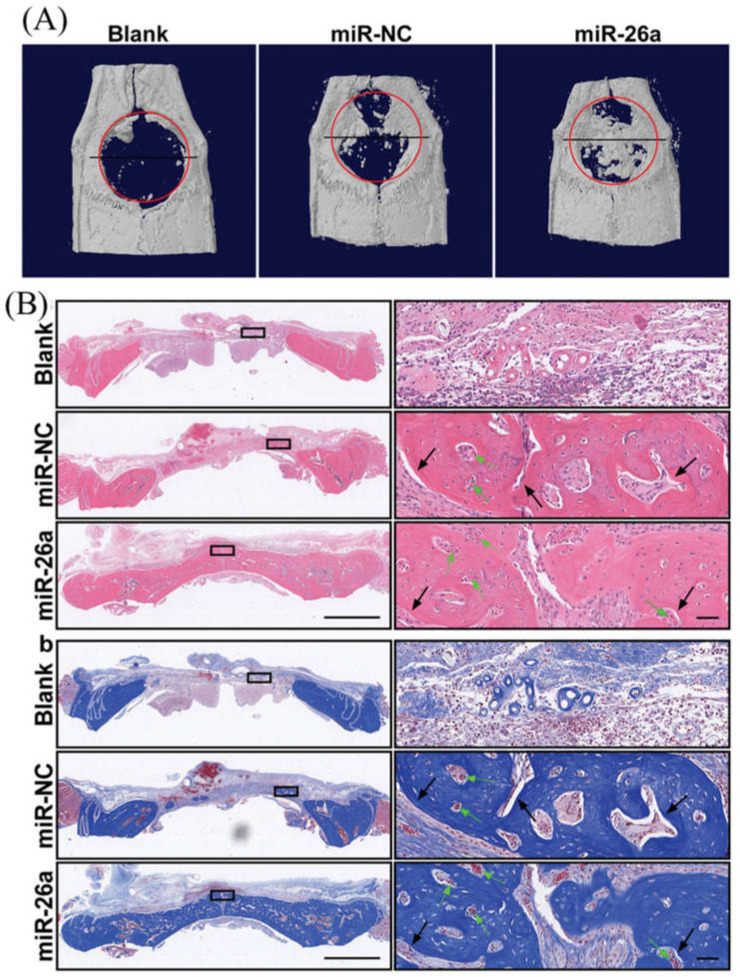
Representative planar radiographs of cranial bone defects at 4 weeks after implantation aerogel nanofibers contain negative control miRNA and miR-26a (**A**), H & E staining, and MTC staining of regenerated tissue after 4 weeks implantation of aerogel nanofibers include negative control miRNA and miR-26a (**B**). The figure is reprinted with permission from reference [99], Copyright 2021, John Wiley and Sons.

Bone tissue engineering builds on the understanding of bone structure and aims to develop platforms that effectively regenerate bone defects. Bone derives its unique mechanical properties from an architectural design extending over nanoscale to macroscopic dimensions. A wide range of structural proteins are found in the ECM of bone, including serum-derived proteins, proteoglycans, glycosylated proteins, Gla-containing proteins, and small integrin-binding proteins. Mentioned proteins bind to strands of collagen fibrils (collagen type I, collagen type X, collagen type III, and collagen type V) [89], dominating at the nanometer level with a diameter scale between 35 to 60 nm. The hydroxyapatite crystals are embedded with collagen molecules and increase the rigidity of the bone. Besides, a wide range of proteins is engaged in the regulation of ECM formation [90]. Bone ECM also contains a number of biologically active molecules. For example, BMP2 [12], IGF1 [91], and TGF-β1 play crucial roles in osteoblast-osteoclast development and bone formation. *RUNX2*, a master transcriptional regulator of osteoblast differentiation [92], responds to numerous extracellular signals, including BMP and TGF-β1, and binds specific DNA sequences with several co-factors to regulate downstream target genes osteoblasts [93]. One such RUNX2 co-factor is SP7, and Sp subfamily member of the Sp/XKLF transcription factor family, leading to the upregulation of type I collagen [94]. SP7 also acts downstream of RUNX2 to regulate osteoblast development [36], exerting its regulatory effects by binding to guanine-rich sequences in specific target genes [95]. Several osteoblast-associated genes/gene products, including *RUNX2*, *SP7*, *ALPL*, *COL1A1*, *IBSP*, and *BGLAP*, are used as markers to demonstrate osteoblastogenesis in a developmental stage-dependent manner [96]. ALPL is a membrane-bound glycosylated enzyme that acts as a phosphatase to release inorganic phosphate from other molecules, increasing the concentration of phosphate required for calcification [97]. As of today, miRNA mimic-NF assemblies with osteogenic activity have been described only in a handful of publications (PubMed) summarizing cell culture models and laboratory animal models. James et al. provided the first report of the potential utility of a miRNA mimic-NF assembly [98]. In this example, electrospun and cross-linked gelatin nanofibers containing miRNA-29a inhibitors were supplied to MC3T3-E1 cells and mouse MSCs. Of the various target candidates tested, Tgfb1 and Igf1 were upregulated by the miRNA-29a inhibitor, leading to increased Sparc in the MC3T3-E1 cells and collagen deposition in MSCs. These NFs (35–50 µm thick) were stable for at least 7 days in the culture medium, and the release of miRNA-29a inhibitor into the medium was found as early as 2 h, gradually increasing and being sustained up to 72 h. To promote the differentiation switch toward osteoblast and accelerate the angiogenesis, osteogenic miR-22 and angiogenic miR-126 were introduced into electrospun PLC NFs [11]. In spite of their almost equal fiber diameter, the NFs derived from miR-22 and miR-26 had less mechanical strength and hydrophobicity. Cell adhesion, proliferation, and osteogenic differentiation of human iPS cells cultured under osteogenic conditions were enhanced by PLC NFs containing miR-22 and miR-26 compared to NFs without these miRNAs. In these cultures, more than 50% of the incorporated miRNAs were released during the first 72 h. Additionally, the 3D nanofiber aerogels containing miR-26a mimic were developed for the regeneration of calvaria bone defects in a rat model. The results of implantations indicate that aerogel- miR-26a mimic promotes bone formation up to 55%, while the aerogel loaded with negative control of miRNA could encourage bone formation up to 20%, as shown in Figure 6. Table 2 represents miRNA mimic-related molecules incorporated into NFs scaffolds for bone regeneration [99].

Two reports have extended our understanding of the effects of miRNA-NFs assemblies on skeletal regeneration in vivo [100,101]. Poly (L-lactic acid) (PLLA)-graft-poly (hydroxyethyl methacrylate) was employed to prepare nanofibrous porous solid microspheres. These solid microspheres with an average diameter between 30 and 60 μM and approximately 15 μM in pore size supported proliferation and chondrogenic differentiation of rabbit MSCs. When rabbit MSCs precultured on solid microspheres carrying antagomir-199a with a hyperbranched polymer vector were implanted into the subcutis of nude mice, and needle puncture-induced lumbar disc degeneration of rabbits, increased chondrogenesis and intervertebral disc regeneration, respectively, were seen. These results were attributed to the antagomir targeting *Hif1a*, with the subsequent upregulation of *Sox9* and other chondrocyte markers. The scaffold carrying hyperbranched polyplex-miR-10a mimic (known as a T regulatory cell [Treg] marker) complexes, in combination with the Treg recruitment factors IL2 and TGFβ (2 mg of microspheres containing 0.8 nmol miR-10a mimic, 1mg IL2, and 2 mg TGFβ protein), served as an injectable scaffold [101]. The nanofibrous scaffold was 60–90 nm in diameter, with multiple pores, and was designed to release IL2, TGFβ, and miR-10a mimic differentially for recruiting Tregs locally and promoting their differentiation effectively. miR-10a was expected to exert its effect on the PI3K-AKT-MTOR pathway by targeting IRS-1 and enhancing Treg differentiation. Since Tregs are a crucial regulator of the immune system in maintaining tolerance to self-antigens and suppressing the activities of other immune cells [102], nanofibrous microspheres carrying the active molecules as above were evaluated in a mouse model of periodontitis, a typical inflammatory disease that leads to gingival recession and alveolar bone defects [103]. Local injection of this assembly into the periodontal margin effectively inhibited the upregulation of gene expression of the primary cytokines of effector T cells in parallel with an increased ratio of Tregs/Th17 cells and concomitantly depressed osteoclastic bone resorption in the foci.

Furthermore, human iPSCs were transduced with miR-2861 mimic, and then the osteogenic differentiation of cells was investigated when cultured on electrospun PLGA nanofibers. Surprisingly, the results indicated that osteogenic functions including *RUNX2*, *ALPL*, *BGLAP*, and *SPARK* in iPSCs improved when the transfected cells were cultured on the NFs and compared to the tissue culture plate. All these indicate the impact of NFs on osteogenic function [104]. Adipose-derived mesenchymal stem cells culturing on miR-181a/b-1 mimic incorporated with PLGA NFs compared with the same cells cultured on the tissue culture plate. The results revealed that adipose-derived mesenchymal stem cells cultured on NFs containing miR-181a/b-1 mimic expressed significantly more *Runx2* and *Spark* than cells cultured on a tissue culture plate [105]. 

## 6. Principal Challenges

Several research studies have shown that nanofibrous scaffolds are useful in tissue engineering, particularly when combined with drugs and other bioactive molecules, including miRNAs, predicting their utility in many important clinical applications to come. [101]. Given our recent findings of selectively stored miRNAs in bone ECM, the development of spatiotemporal-specific delivery platforms for miRNAs may benefit from an ability to harness the biomimetic properties of the skeletal tissue itself. Although further research will be required to address fundamental issues, such as ways to select the most advantageous miRNAs for skeletal tissue engineering. Considering a cocktail of multiple miRNAs, as discussed above [106], it may not only be the most advantageous, but also required for the desired outcome. To this end, an essential early step will be to develop a comprehensive miRNA picture of the bone microenvironment during regeneration. Furthermore, we believe that future approaches that incorporate miRNAs may use multifunctional nano scaffolds incorporating nanomaterials into miRNA-NF assemblies to control miRNA delivery more accurately and efficiently. Such a delivery system was the case when an NF platform coupling graphene oxide to gold nanoparticles carrying miR-101 had been used for the higher near-infrared thermal therapy of breast cancer. The prepared scaffold was induced cell death; not only by increasing the temperature inside cells, but also by the miR-101-dependent induction of apoptosis [107]. These biologically inspired nanomaterials may benefit patients with bone defects due to bone metastasis, for example. 

Addressing all the issues that need to be considered, such as optimizing the release duration based on instability of miRNAs, improving the cellular uptake, and tissue-specific delivery to avoid systemic toxicity and off-target effect, delivery platforms, as well as NFs, will continue to be promising carriers for miRNAs.

## Figures and Tables

**Table 1:** miRNAs incorporated with hydrogel scaffolds for bone tissue engineering.

miRNAs	Target Cell or Animal	Target Genes	Refs
In vitro studies			
Let-7a	Human USSC	*NLK*	[27]
Let-7f	Human MSCs	*AXIN2*	[28]
miRNA-15b	Human MSCs	*BMPR2*	[29]
miRNA-20a	Human MSCs	*PPARG, BAMBI, CRIM1*	[30]
miRNA-27	Human FOB1.19 cells	*APC*	[31]
miRNA-29a/b/c	Mouse primary osteoblast, MC3T3 cells	*Bglap, Acvr2a, Ctnnbip, Dusp2, Hdac4, Tfgb3*	[32,33,34]
miRNA-130c	Human MSCs	*CAMTA1, CXCL12, ITGB1, FLT1*	[29]
miRNA-96	Human MSCs	*FABP4*	[35]
miRNA-130b	Human MSCs	*CAMTA1, CD44, GDF6, PDGFRA, COL9A3*	[29]
miRNA-142-3p	Human FOB1.19 cells	*APC*	[36]
miRNA-199a	Human FOB1.19 cells	*SOX9*	[35]
miRNA-210	Mouse ST2 cells	*Acvr1b*	[37]
miRNA-218	Mouse MSCs	*Dkk2, Sfrp2, Sost*	[38]
miRNA-355-5p	MC3T3-E1, MLO-A5 cells	*Dkk1*	[39]
miRNA-1228	Human primary osteoblast	*BMP2K*	[40]
miRNA-2861/miRNA-3960	Mouse ST2 cells, Mouse osteoblast	*Hdac4, Hoxa2*	[41]
Anti-miRNA-133a	Human MSCs	*RUNX2*	[42]
miRNA-16	Human MSCs	*RUNX2*	[43]
miRNA-590-5p	C3H10T1/2	*Smad7*	[44]
miRNA-122	Human MSCs	*RUNX2, OSX*	[45]
In vivo studies			
Anti-miRNA-214	Ovariectomy, mouse	*Atf4*	[46]
Anti-miRNA -92a	Femoral fracture, mouse	*Itga5, Mkk4*	[47]
Anti-miRNA -31	Calvarium defect, rat and canine	*Satb2*	[48]
Anti-miRNA -26a	Calvarium defect, mouse	*Ptn*	[49]
Anti-miRNA -138	Subcutaneous, mouse	*Runx2, Sp7, Bmp2*	[50]
miRNA-148b mimic	Calvarium defect, mouse	*Runx2*	[51]
miRNA-5106	Calvarium defect, mouse	*Runx2, Spp1, Alpl*	[52]
miRNA-34a	Tibial defect, rat	*Notch1*	[53]
Anti-miRNA-222	The refractory fracture, rat	*Col2a1, SOX9*	[54]
Anti-miRNA-432	Ectopic bone formation, mouse	*Cdkn1b*	[55]

**Table 2:** miRNA mimic-related molecules incorporated into NFs.

NF	miRNA	Target Gene(s)	Model/Application	Ref
Crosslinked gelatin	TransIT-TKO^®^-Antagomir-29a complex	*Tgfb1, Igf1*	Mouse MC3T3E1 cells, mouse MSCs in vitro	[98]
PLC-gelatin NF	Polyplex-miR-22 and miR-26 mimic complex	*RUNX2, SPARC, BGLAP*	Human iPS cells in vitro	[11]
PLL-PHN-NF-SMS	HP-antagomir-199a complex	*Hif1a*	Rabbit MSCs in vitro, subcutaneous implantation in nude mice, implantation into an intervertebral disc degeneration rabbit model	[100]
PLLA-NF-SMS	HP-miR-10a mimic complex	*Irs1*	Mouse T cells, injection of complex plus TGFβ1 into a periodontitis mouse model	[101]
PLGA	miR-2861 mimic	*RUNX2*	Human iPS cells, in vitro	[104]
PEG/PLGA	miR-181a/b-1	*PTEN/PI3KK/AKT*	Adipose-drived mesenchymal stem cells, in vitro	[105]
HA-SS-PGEA	miR-26a	*Runx2, Alpl, Bglap, and Bsp*	Rat MSCs, rat calvaria defect model	[99]

## Data Availability

The schematics and illustrations are made by Blender 2.93.6.

## Supplementary Materials

The following are available online at https://www.mdpi.com/article/10.3390/mi12121472/s1, Table S1: The symbols and abbreviations.
