# Supplementary material for: MiRNA-Nanofiber, the Next Generation of Bioactive Scaffolds for Bone Regeneration: A Review
Source: Micromachines (Basel). 2021 Nov 29;12(12):1472. doi: 10.3390/mi12121472 (PMC8707075; doi:10.3390/mi12121472)
Supplement: Supplementary file 1 [file micromachines-12-01472-s001.zip › micromachines-1473644-supplementary.pdf]

Supplementary data

Micro RNA- nanofiber, next generation of bioactive scaffolds for bone regeneration: a review

Table S1. The symbols and abbreviations.

Symbole or abbreviation	Description
**2D**	Two-dimensional
**3D**	Three-dimensional
**ACVR1B**	Activin A Receptor Type 1B
**ACVR2A**	Activin A Receptor Type 2A
**AKT**	AKT Serine/Threonine Kinase
**AKT1**	AKT Serine/Threonine Kinase Type 1
**ALPL**	Alkaline Phosphatase, Biomineralization Associated
**Antagomirs**	miRNA inhibitors
**APC**	APC Regulator Of WNT Signaling Pathway
**ATF4**	Activating Transcription Factor 4
**BAMBI**	BMP And Activin Membrane Bound Inhibitor
**BCL2**	BCL2 Apoptosis Regulator
**BGLAP**	Bone Gamma-Carboxyglutamate Protein
**BMP**	Bone Morphogenic Protein
**BMP2K**	BMP2 Inducible Kinase
**BMPR2**	Bone Morphogenetic Protein Receptor Type 2
**CAMTA1**	Calmodulin Binding Transcription Activator 1
**CDKN1B**	Cyclin Dependent Kinase Inhibitor 1B
**CELF2**	CUGBP Elav-Like Family Member 2
**COL1A1**	Collagen Type I Alpha 1 Chain
**COL1A2**	Collagen Type 1 alpha 2 Chain
**COL9A3**	Collagen Type 9 Alpha 3 Chain
**CRIM1**	Cysteine Rich Transmembrane BMP Regulator 1
**CTNNBIP**	Catenin Beta 1
**CXCL12**	C-X-C Motif Chemokine Ligand 12
**CXCR4**	C-X-C Motif Chemokine Receptor 4
**DDS**	Drug Delivery Systems
**DKK**	Dickkopf WNT Signaling Pathway Inhibitor
**DNPEP**	Asparty Aminopeptidase
**DUSP2**	Dual Specificity Phosphatase 2
**ECM**	Extracellular matrix
**FABP4**	Fatty Acid Binding Protein 4
**FGF2**	Fibroblast Growth Factor 2
**FLT1**	Fms Related Receptor Tyrosine Kinase 1
**GDF6**	Growth Differentiation Factor 6

**HDAC4**	Histone Deacetylase 4
**HIF1A**	Hypoxia Inducible Factor 1 Subunit Alpha
**HOXA2**	Homeobox A2
**IBSP**	Integrin Binding Sialoprotein
**IGF1**	Insulin Like Growth Factor 1
**IL10**	Interleukin 10
**IL1A**	Interleukin 1 Alpha
**IL1B**	Interleukin 1 Beta
**IL2**	Interleukin 2
**IRS1**	Insulin Receptor Substrate 1
**ITGA5**	Integrin Subunit Alpha 5
**ITGB1**	Integrin Subunit Beta 1
**MAP2K4**	Mitogen-Activated Protein Kinase Kinase 4
**miRNA**	micro RNAs
**miRNA mimic**	Synthetic micro RNA
**mRNAs**	Messenger RNAs
**MSCs**	Bone marrow mesenchymal stem cells
**MTOR**	Mechanistic Target Of Rapamycin Kinase
**NCOA1**	Nuclear Receptor Coactivator 1
**NFs**	Nanofibers
**NLK**	Nemo Like Kinase
**NOTCH1**	Notch Receptor 1
**NRIP1**	Nuclear Receptor Interacting Protein 1
**PCL**	Polycaprolactone
**PCL/SF**	Poly(ε-caprolactone)/silk
**PDGFRA**	Platelet derived growth factor receptor alpha
**PGMA**	Poly (glycidyl methacrylate
**PI3K**	Phosphatidylinositol 3-kinase, putative
**PLGA**	Poly (lactic-co-glycolic acid)
**PLLA**	Poly (L-lactic acid)
**PPARG**	Peroxisome Proliferator Activated Receptor Gamma
**PTEN**	Phosphatase and Tensin Homolog
**RALA**	RAS Like Proto-Oncogene A
**RUNX2**	RUNX Family Transcription Factor 2
**Satb2**	SATB Homeobox 2
**SFRP2**	Secreted Frizzled Related Protein 2
**SMAD**	SMAD Family Member
**SOST**	Sclerostin
**SOX9**	SRY-Box Transcription Factor 9
**SP7**	Sp7 Transcription Factor
**SPARK**	Secreted Protein Acidic And Cysteine Rich
**SPP1**	Secreted Phosphoprotein 1

**TGFBR1**	Transforming Growth Factor Beta Receptor 1
**TGFB**	Transforming Growth Factor Beta
**Th1 cytokines**	Stimulate macrophages, lymphocytes, and PMNs
**TLR4**	Toll Like Receptor 4
**TNF**	Tumor Necrosis Factor
**VEGFA**	Vascular Endothelial Growth Factor A
